# Current-Volt Biosensing “Cystatin C” on Carbon Nanowired Interdigitated Electrode Surface: A Clinical Marker Analysis for Bulged Aorta

**DOI:** 10.1155/2022/8160502

**Published:** 2022-05-23

**Authors:** Xi Chen, Jie Kang, Qiu Sun, Cheng Liu, Hongling Wang, Chen Wang, Subash C. B. Gopinath

**Affiliations:** ^1^Department of Vascular Surgery, Wuhan No.1 Hospital, WuHan, HuBei 430022, China; ^2^Department of Vascular Surgery, Liaocheng People's Hospital, Liaocheng, Shandong Province 252000, China; ^3^Department of Intervention, Wuhan No. 1 Hospital, Wuhan, Hubei 430022, China; ^4^Department of Vascular Surgery, Nanjing Drum Tower Hospital, The Affiliated of Nanjing University Medical School, Nanjing City, Jiangsu Province 730050, China; ^5^Department of Cardiothoracic Surgery, Hospital of Lianqin Security Force 940th, Lanzhou, Gansu 730000, China; ^6^Department of Peripheral Vascular Intervention, Gansu Provincial Hospital of TCM, No. 418 Guazhou Road, Qilihe District, Lanzhou City, Gansu Province 730050, China; ^7^Institute of Nano Electronic Engineering, Universiti Malaysia Perlis (UniMAP), Kangar 01000, Perlis, Malaysia; ^8^Faculty of Chemical Engineering Technology, Universiti Malaysia Perlis (UniMAP), Arau 02600, Perlis, Malaysia; ^9^Centre of Excellence for Nanobiotechnology and Nanomedicine (CoExNano), Faculty of Applied Sciences, AIMST University, Semeling 08100, Kedah, Malaysia

## Abstract

A carbon nanowire-modified surface with interdigitated electrode (IDE) sensing system was introduced to identify abdominal aortic aneurysm biomarker “papain,” also known as cysteine protease, used as the capture probe to identify Cystatin C. Papain was immobilized through the covalent integration of amine group on papain and the carboxyl group with carbon nanowire. This papain-modified electrode surface was utilized to detect the different concentrations of Cystatin C (100 pg/mL to 3.2 ng/mL). The interaction between papain and Cystatin C was monitored using a picoammeter, and the response curves were compared. With increasing Cystatin C concentrations, the total current levels were gradually increased with a linear range from 200 pg/mL to 3.2 ng/mL, and the current differences were plotted and the detection limit of Cystatin C was calculated as 200 pg/mL. The averaging of three independent experiments (*n* = *3*) was made with 3*δ* estimation, and the determination coefficient was *y* = 1.8477 × 0.7303 and *R*^2^ = 0.9878. Furthermore, control experiments with creatinine and gliadin failed to bind the immobilized papain, indicating the specific detection of Cystatin C.

## 1. Introduction

Abdominal aortic aneurysm (AAA) is a fatal arterial disease, defined by an abnormal bulge in the artery wall. AAA is generally developed by the weakened artery due to injury, infection, or congenital defect in the connective tissue of the artery [1, 2]. Various other reasons also contribute to AAA, which include genetic susceptibility, atherosclerosis, smoking, and hypertension [3, 4]. AAA is generally developed in the abdominal aorta between the renal and iliac arteries [5], and a diameter of >3 cm causes various health issues [6]. In particular, if the aneurysm size expands above 5.5 cm, it ruptures and causes bleeding which leads to death [2]. AAA is a dynamic and complex pathophysiological process, and the molecular mechanism of AAA is still unclear. At present, open surgical repair and invasive endovascular aneurysm repair are the possible treatments for AAA. Diagnosing the condition and size of AAA at its earlier stage helps to treat the patient before rupturing. The rupturing of AAA is asymptomatic, and the present imaging methods, such as ultrasound computerized tomography and magnetic resonance imaging, are helpful in identifying AAA, but these methods are inconvenient and expensive. So, it is mandatory to develop a sensing strategy to diagnose AAA by blood-based biomarker analysis [2, 7–9]. Various blood-based biomarkers help to identify and diagnose the disease at its earlier stages, which helps to provide the proper treatment at the right time [10–13]. In this research work, Cystatin C was used as the biomarker to diagnose AAA.

Cystatin C is the endogenous inhibitor of the elastolytic enzymes cathepsins, which are expressed ubiquitously in most of the tested cells. Research has found that the expression of Cystatin C is reduced in patients with atherosclerotic lesions and AAA. Furthermore, the Cystatin C level was significantly lower in AAA patients than in patients with non-AAA [14, 15], and it was further noticed that there is a negative correlation of serum Cystatin C with the size of AAA and its annual expansion rate [16]. So, quantifying cystatin helps to diagnose AAA with its size changes. Various diagnosing systems have been developed for cysteine identification, but those methods are time consuming, tedious, and expensive and need sophisticated equipment, as well as a well-trained manpower. This research focused to detect Cystatin C by using the cysteine protease/papain as the capture probe to diagnose AAA on sensing surface by interdigitated electrode (IDE). IDE sensing surface has dual electrodes, which form equally gapped regions that can be connected to a probe station. Upon attaching/interacting the molecules on the gap or electrode regions, there will be a creation of molecular vibrations [17–19]. Ultimately, a dipole moment forms among the ions, which change the output by the transducer.

Biosensors have been categorized by the type of transducer used, such as colorimetric, electrochemical, piezoelectric, and fluorescence biosensors [20–23]. Among them, current-volt biosensors have become popular and attractive due to its higher sensitivity, easy handling, ability to collect data rapidly, and higher selectivity [24–27]. Further modification on electrode surface with nanomaterials increases the surface area, high-probe immobilization, and higher electron transfer kinetics [28–32]. Herein, we introduced a carbon nanowire-modified electrode to immobilize papain, and it increases the electric flow [33–35]. A carboxyl functionalized nanowire was attached on the potassium hydroxide-treated electrode surface, and then, the amine group on papain was attached covalently on the nanowire. The primary advantage of the carbon nano-wired interdigitated electrode is to enhance the surface area. Ultimately, a higher molecular immobilization/assembly will occur with Cystatin C interaction. With the enhanced surface area, there is a potential dipole moment between the two electrodes, which will substantiate the changes on the surface upon molecular attachment or interaction. This electrode was used as the working surface to monitor the protein-protein interaction with papain and Cystatin C, and the transduction of electrons was monitored by current-volt measurements. The response curves were recorded with a picoammeter to monitor the papain and Cystatin C interaction in a dose-dependent manner.

## 2. Experimental

### 2.1. Materials

The electrode surface was obtained from Metrohm (Switzerland) for interdigitated electrode (IDE) analysis. Cystatin C, papain, N-hydroxysuccinimide (NHS), phosphate buffer saline (PBS), and ethyl-3-(3-dimethyl-aminopropyl) carbodiimide (EDC), potassium hydroxide (KOH), and serum albumin were ordered from Sigma Aldrich (Missouri, USA).

### 2.2. Electrode Surface Functionalization

The IDE surface was modified into a carbon nanowire for attaching the capture probe, papain. Initially, the electrode surface was cleaned with distilled water and then treated with diluted (1%) KOH for 7 min followed by washing with distilled water to eliminate the unbound KOH. After that, 1% of diluted APTES in ethanol was added on the surface and kept overnight. The next day, the APTES-coated electrode surface was cleaned with 30% of ethanol thoroughly to remove the unbound APTES. And then, 1 g of carbon nanowire was dispersed and dropped on the APTES-modified electrode and kept for 1 h and then washed with PBS buffer. Furthermore, the electrode surface was incubated in EDC and NHS mixture (10 mM, 1 : 1 v/v in PBS) for 1 h. Excess EDC-NHS was washed off with PBS, and then, 2 mg/mL of diluted papain was dropped and allowed to rest for 1 h to allow the papain binding on carbon nanowire. This papain modified electrode was utilized to quantify the Cystatin C level by using current-volt measurements.

### 2.3. Cystatin C Identification on Papain-Modified Electrode Surface

Cystatin C was detected on the carbon nanowire-papain-modified IDE surface. To get the specific interaction of Cystatin C and papain, the uncovered activated carbon nanowire was covered with 1% PEG-NH_2_ of diluted PBS for 30 min. And then, different concentrations of 100, 200, 400, 800, 1600, and 3200 pg/mL of Cystatin C were dropped individually on the papain-immobilized electrode surface and placed for 30 min. After washing the surface with PBS, the electric signal was measured. The response curves by current-volt measurements were recorded to monitor papain and Cystatin C interaction.

### 2.4. Specificity and Reproducibility of the Papain-Modified Electrode

Control experiments with creatinine and gliadin were conducted to identify the specific detection of Cystatin C on the papain modified electrode surface. For this experiment, control proteins at 3.2 ng/mL were added on a papain immobilized electrode and the electric signal was recorded. Furthermore, a control experiment was also conducted without papain. For this experiment, Cystatin C was placed on carbon nanowire (without papain) and then the current flow was recorded. The response cure was compared with the specific binding of Cystatin C with papain.

Furthermore, to confirm the reproducibility of the papain-modified electrode, the same experiments were conducted with five different batches of electrodes and the current response for each immobilization; APTES, CNW, papain, PEG-NH_2_ and Cystatin C were recorded for comparison. All experimental conditions were at room temperature and with a wet sensing surface. Washings were performed between each surface modification or interaction. The current was given from 0–2 V at an interval of 0.1 V which was used in the whole experiment.

## 3. Results and Discussion

**Figure 1:**
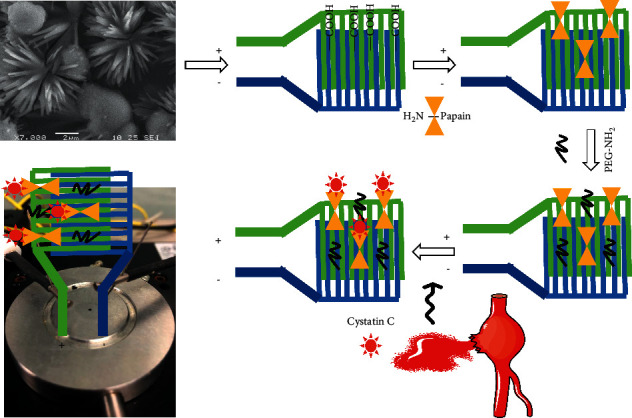
Schematic for surface modifications/functionalization. The different steps involved are shown. A dual probe station is displayed. The physical appearance of the aorta is also shown. The nanowire on the surface was evidenced by scanning electron microscope observation.

**Figure 2:**
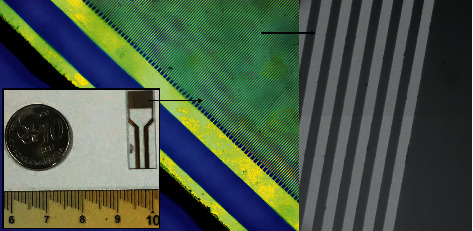
Surface of an interdigitated electrode sensor observed under both a high-power microscope (blue) and a 3D-nanoprofiler (grey). The figure inset shows the original sensing device.

**Figure 3:**
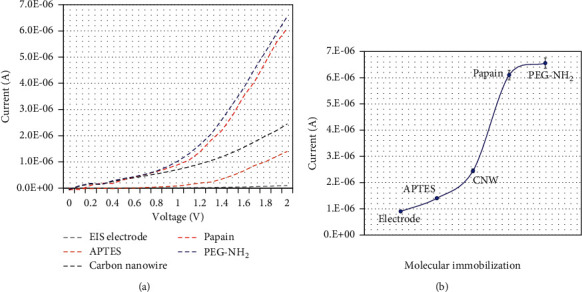
Process of the capture probe “papain” immobilization on the carbon nanofiber-modified interdigitated electrode surface. (a) Voltages vs current responses for the immobilization process. Clear increment of current was noted after each immobilization. (b) The current level of the capture probe “papain” immobilization process. A higher increment of current was recorded after adding papain on the electrode.

The abdominal aortic aneurysm (AAA) biomarker, “Cystatin C,” was detected on the carbon nanowire-modified electrode surface.  Figure 1 displays the schematic illustration of Cystatin C detection on the papain modified electrode surface. Carbon nanowire was evidenced by scanning electronic microscope imaging (Figure 1; inset) and immobilized on the electrode surface through APTES as a linker, and then, papain was linked with carbon nanowire through covalent bonding. And then, PEG-NH2 was utilized here as the blocking agent to avoid the nonspecific Cystatin C binding on the electrode surface. The changes in the current supplied with the ammeter were recorded to monitor the biological interaction on the IDE surface. Before initiating the experiment, the intactness of sensing was confirmed with a high-power microscope and 3D-nanoprofiler (Figure 2).  Figure 3 shows the process of the capture probe (papain) immobilization on the carbon nanofiber-modified IDE surface.  Figure 3(a) shows the relationship between voltage and current for the immobilization process. The bare electrode shows the current response as 9.0 E-07 A. After modifying the electrode surface by APTES, the response was elevated to 1.4 E-06 A. This result confirms the aminated surface with the IDE surface. Furthermore, upon adding the carbon nanowire, response was increased to 2.45 E-06 A, and when the capture probe of papain was dropped, the response was drastically increased to 6.1 E-06 A. This enhancement confirms the attachment of papain with the immobilized carbon nanowire. Finally, the blocking molecule, PEG-NH_2_, was added on the papain modified electrode surface, and there were no significant changes in response. This might be due to the high occupancy of papain on the carbon nanowire surface (Figure 3(b)).

### 3.1. Detection of Cystatin C on the Papain-Immobilized Electrode Surface

**Figure 4:**
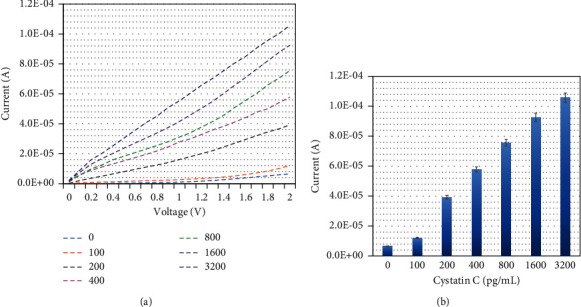
Detection of different concentrations of cystatin C by the immobilized papain surface. (a) The Cystatin C and papain interaction was analyzed by voltage vs current responses. The clear increment of current responses confirms the interaction of Cystatin C with the immobilized papain. (b) The current level of Cystatin C and papain interaction increases the Cystatin C concentration, and the response also increases gradually.

**Figure 5:**
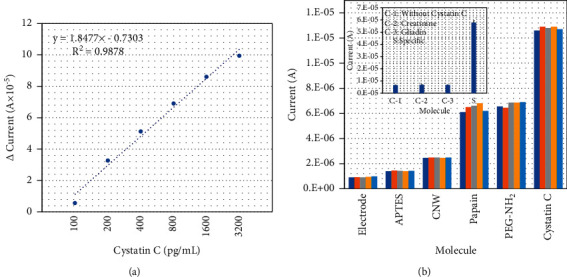
(a) Differences in current response (from blank) for different concentrations of Cystatin C were plotted in an excel sheet, and the limit of Cystatin C detection was calculated in the range between 100 and 200 pg/mL with the *R*^2^ value of 0.9878. (b) Reproducibility analysis of Cystatin C identification. There are no significant changes in the current response of the bare electrode, APTES, CNW, papain, PEG-NH_2_, and Cystatin C with five different electrodes, which confirms the reproducibility of the papain-modified electrode. The figure inset displays the specificity analysis of Cystatin C control experiments with creatinine and gliadin (instead of Cystatin C) conducted to identify the specific detection of Cystatin C on the papain-modified electrode surface. Without Cystatin C and with control protein, no significant changes of current response were noted. Cystatin C was specifically recognised by the immobilized papain.

Different levels of Cystatin C were dropped on a constant papain-modified electrode surface. The changes in the current output were recorded to monitor the interaction of papain with Cystatin C (Figure 4(a)). Figure 4(a) shows the relationship between voltage and current for the interaction of papain with different concentrations of Cystatin C. With zero Cystatin C, the current response was recorded as 6.55 E-06 A; when 100 pg/mL of Cystatin C was dropped, the response was increased to 1.22 E-05 A. This small alteration is due to the interaction of papain with Cystatin C. Further increment in Cystatin C concentrations to 200, 400, 800, 1600, and 3200 pg/mL, responses were gradually increased to 3.93, 5.78, 7.56, 9.26, and 10.6 E-05 A, respectively. It was clearly noted that with increasing Cystatin C concentration, the current responses were also gradually enhanced (Figure 4(b)) and the difference in response for each Cystatin C interaction with papain was calculated and plotted in an excel sheet. The limit of detection of Cystatin C was calculated at the level of 200 pg/mL with the *R*^2^ value of 0.9878 (Figure 5(a)).

### 3.2. Reproducibility and Specificity of the Papain-Modified Electrode Surface

Control experiments with creatinine and gliadin (instead of Cystatin C) were conducted to identify the specific detection of Cystatin C on the papain-modified electrode surface. As in Figure 5(b) (inset), without Cystatin C and with control protein, no significant changes of current response were noted. At the same time, with specific interaction of papain and Cystatin C being recorded, it is confirmed that Cystatin C was specifically recognised by the immobilized papain.

Furthermore, to confirm the reproducibility of the papain-modified electrode, the same experiments were conducted with five different batches of electrodes. As shown in Figure 5(b), there were no significant changes of current response of bare electrode, APTES, CNW, papain, PEG-NH_2_, and the cystatin C with different electrodes. This result confirms the reproducibility of the papain-modified electrode. This system is quite feasible with the same operating set-up for analysing clinically relevant real samples. With the similar sensing system on different modifications, the genuine detections have been demonstrated as the evidence using human serum [22, 36, 37]. According to these recent studies, the primary optimization step is necessary with different surface modifications to be suited for the desired measurement ranges.

## 4. Conclusion

Abdominal aortic aneurysm (AAA) is defined by an abnormal bulge in the artery wall and the burst with AAA cause excess bleeding, which is fatal. This research work designed a sensing system to detect AAA biomarker, “Cystatin C” with the cysteine protease, papain, by the current-volt measurements. To immobilize the papain, the carbon nanowire was attached on the electrode through amine modification, and then, papain was attached on through the covalent binding. Different cystatin C ranges were detected on the papain-modified electrode surface, and the limit of detection was found at 200 pg/mL. In addition, control proteins did not show any significant current increment, indicating the specific detection of Cystatin C. This study provides information for the identification of Cystatin C and generates the sensing platform with other clinical biomarkers.

## Data Availability

Data used to support the findings of this study are available upon request.

## References

[1] Springer F., Schlierf R., Pfeffer J. G., Mahnken A. H., Schnakenberg U., Schmitz-Rode T. (2007). Detecting endoleaks after endovascular AAA repair with a minimally invasive, implantable, telemetric pressure sensor: an in vitro study. *European Radiology*.

[2] Guo S., Li Y., Li R. (2020). High-performance detection of an abdominal aortic aneurysm biomarker by immunosensing. *Biotechnology and Applied Biochemistry*.

[3] Tambyraja A. L., Chalmers R. T. A. (2009). Aortic aneurysms. *Surgery*.

[4] Danzer D., Becquemin J. P. (2018). Abdominal aortic aneurysm. *Vascular Surgery: Cases, Questions and Commentaries*.

[5] Doron E., Penner A. (2003). Acoustic biosensor for monitoring physiological conditions in a body implantation site. *Journal of the Acoustical Society of America*.

[6] Maguire E. M., Pearce S. W. A., Xiao R., Oo A. Y., Xiao Q. (2019). Matrix metalloproteinase in abdominal aortic aneurysm and aortic dissection. *Pharmaceuticals*.

[7] Soto B., Gallastegi-Mozos T., Rodriguez C. (2017). Circulating CCL20 as a new biomarker of abdominal aortic aneurysm. *Scientific Reports*.

[8] Ding Y., Tian Q., Dong Y., Xing L., Gopinath S. C. B., Mao Y. (2021). Gold-silane complexed antibody immobilization on polystyrene ELISA surface for enhanced determination of matrix Metalloproteinase-9. *Process Biochemistry*.

[9] Hong X., Zhao H., Chen C. (2020). Improved immunoassay for insulin-like growth factor 1 detection by aminated silica nanoparticle in ELISA. *Process Biochemistry*.

[10] Montano N., D’Alessandris Q. G., Izzo A., Fernandez E., Pallini R. (2016). Biomarkers for glioblastoma multiforme: status quo. *Journal of Clinical and Translational Research*.

[11] Mcgill M. R., Jaeschke H. (2021). Biomarkers of mitotoxicity after acute liver injury: further insights into the interpretation of glutamate dehydrogenase. *Journal of Clinical and Translational Research*.

[12] Liu Y. (2021). Research progress of diabetes mellitus complicated with cardiovascular disease. *Journal of Clinical and Nursing Research*.

[13] Yan L., Liu Q., Zhang C. (2021). Progress in diagnosis and endoscopic treatment of stanford *B* penetrating aortic ulcers. *Journal of Clinical and Nursing Research*.

[14] Lindholt J. S., Erlandsen E. J., Henneberg E. W. (2002). Cystatin C deficiency is associated with the progression of small abdominal aortic aneurysms. *British Journal of Surgery*.

[15] Shi G. P., Sukhova G. K., Grubb A. (1999). Cystatin C deficiency in human atherosclerosis and aortic aneurysms. *Journal of Clinical Investigation*.

[16] Lv B. J., Lindholt J. S., Cheng X., Wang J., Shi G. P. (2012). Plasma cathepsin s and cystatin c levels and risk of abdominal aortic aneurysm: a randomized population-based study. *PLoS One*.

[17] Yu Z., Cai G., Liu X., Tang D. (2021). Pressure-based biosensor integrated with a flexible pressure sensor and an electrochromic device for visual detection. *Analytical Chemistry*.

[18] Zeng R., Wang W., Chen M. (2021). CRISPR-Cas12a-driven MXene-PEDOT:PSS piezoresistive wireless biosensor. *Nano Energy*.

[19] Chen J., Tong P., Huang L., Yu Z., Tang D. (2019). Ti_3_C_2_ MXene nanosheet-based capacitance immunoassay with tyramine-enzyme repeats to detect prostate-specific antigen on interdigitated micro-comb electrode. *Electrochimica Acta*.

[20] Lakshmipriya T., Fujimaki M., Gopinath S. C. B., Awazu K., Horiguchi Y., Nagasaki Y. (2013). A high-performance waveguide-mode biosensor for detection of factor IX using PEG-based blocking agents to suppress non-specific binding and improve sensitivity. *Analyst*.

[21] Wang F. A., Lakshmipriya T., Gopinath S. C. B. (2018). Red spectral shift in sensitive colorimetric detection of tuberculosis by ESAT-6 antigen-antibody complex: a new strategy with gold nanoparticle. *Nanoscale Research Letters*.

[22] Yao J., Li S., Zhang L. (2020). Aptamer-antibody dual probes on single-walled carbon nanotube bridged dielectrode: comparative analysis on human blood clotting factor. *International Journal of Biological Macromolecules*.

[23] Lakshmipriya T., Fujimaki M., Gopinath S. C. B., Awazu K. (2013). Generation of anti-influenza aptamers using the systematic evolution of ligands by exponential enrichment for sensing applications. *Langmuir*.

[24] Ramanathan S., Gopinath S. C. B., Arshad M., Poopalan P. (2019). Multidimensional (0D–3D) nanostructures for lung cancer biomarker analysis: comprehensive assessment on current diagnostics. *Biosensors and Bioelectronics*.

[25] Ramanathan S., Jusoh M., Sabapathy T. (2020). Elastomeric polydimethylsiloxane polymer on conductive interdigitated electrode for analyzing skin hydration dynamics. *Applied Physics A*.

[26] Kazemi S. H. K., Ghodsi E., Abdollahi S., Nadri S. (2016). Porous graphene oxide nanostructure as an excellent scaffold for label-free electrochemical biosensor: detection of cardiac troponin I. *Materials Science and Engineering: C*.

[27] Wang Q., Zhou Z., Zhai Y. (2015). Label-free aptamer biosensor for thrombin detection based on functionalized graphene nanocomposites. *Talanta*.

[28] Carrasco-esteban E., Domínguez-rullán J. A., Barrionuevo-castillo P., Pelari-mici L., Leaman O., Sastre-Gallego S. (2021). Current role of nanoparticles in the treatment of lung cancer. *Journal of Clinical and Translational Research*.

[29] Zhang Y., Attarilar S., Wang L., Lu W., Yang J., Fu Y. (2021). A review on design and mechanical properties of additively manufactured NiTi implants for orthopedic applications. *International Journal of Bioprinting*.

[30] Lu B., Liu L., Wang J. (2020). Detection of microRNA-335-5p on an interdigitated electrode surface for determination of the severity of abdominal aortic aneurysms. *Nanoscale Research Letters*.

[31] Curulli A., Cesaro S. N., Coppe A., Silvestri C., Palleschi G. (2006). Functionalization and dissolution of single-walled carbon nanotubes by chemical-physical and electrochemical treatments. *Microchimica Acta*.

[32] Chen S., Jang T. S., Pan H. M. (2020). 3D freeform printing of nanocomposite hydrogels through in situ precipitation in reactive viscous fluid. *International Journal of Bioprinting*.

[33] Hou Y., Wang W., Bartolo P. (2020). Investigating the effect of carbon nanomaterials reinforcing poly(Ε-caprolactone) scaffolds for bone repair applications. *International Journal of Bioprinting*.

[34] Shuai C., Li Y., Yang W. (2020). Graphene oxide induces ester bonds hydrolysis of poly-l-lactic acid scaffold to accelerate degradation. *International Journal of Bioprinting*.

[35] Rueda-Gensini L., Serna J. A., Cifuentes J., Cruz J. C., Muñoz-Camargo C. (2021). Graphene oxide-embedded extracellular matrix-derived hydrogel as a multiresponsive platform for 3D bioprinting applications. *International Journal of Bioprinting*.

[36] Li Z., Gopinath S. C. B., Lakshmipriya T., Anbu P., Perumal V., Wang X. (2020). Self-assembled silver nanoparticle-DNA on a dielectrode microdevice for determination of gynecologic tumors. *Biomedical Microdevices*.

[37] Yu Z., Gopinath S. C. B., Lakshmipriya T., Anbu P. (2021). Single-walled carbon nanotube-gold urchin nanohybrid for identifying gastric cancer on dimicroelectrodes junction. *Journal of the Taiwan Institute of Chemical Engineers*.

